# Burden of musculoskeletal disorders among registered nurses: evidence from the Thai nurse cohort study

**DOI:** 10.1186/s12912-017-0263-x

**Published:** 2017-11-21

**Authors:** Wilaiphorn Thinkhamrop, Krisada Sawaengdee, Viroj Tangcharoensathien, Tuangtip Theerawit, Wongsa Laohasiriwong, Jiamjit Saengsuwan, Cameron Paul Hurst

**Affiliations:** 1 Faculty of Public Health, Khon Kaen University, Khon Kaen, 40002 Thailand; 2 International Health Policy Program, Ministry of Public Health, Nonthaburi, 11000 Thailand; 3 Faculty of Public Health, Board Committee of Research and Training Centre for Enhancing Quality of Life of Working Age People (REQW), Khon Kaen University, Khon Kaen, 40002 Thailand

**Keywords:** Musculoskeletal disorders, Cohort study, Registered nurse, Burden, Prevalence

## Abstract

**Background:**

Musculoskeletal disorders (MSDs) are a major public health problem among registered nurses (RNs) in Thailand. Information on their burdens at a national level is limited. This study estimated the prevalence of MSDs among RNs using the 2009 Thai Nurse Cohort, a nationally representative sample of RNs in Thailand.

**Methods:**

This study is part of the first wave survey of the Thai Nurse Cohort Study (TNCS) conducted in 2009. Members of the cohort consisted of 18,756 RNs across Thailand. A 13-page self-administered questionnaire was sent to participants where MSDs were measured by self-reported answers to questions related to experiencing MSDs during a previous year. However, 1070 RNs were excluded from this study since they were unemployed during a previous year, therefore the final sample size was 17,686 RNs. A 12-month prevalence of MSDs and its 95% confidence interval (95% CI) were estimated based on normal approximation to binomial distribution. Chi-square test for trend was used.

**Results:**

Of the 17,686 RNs, 47.8% (95% CI: 47.0–48.5) reported having MSDs during the previous 12 months. The prevalence of MSDs significantly increased with age, body mass index, and working duration (all *P* < 0.001). Compared to the non-MSD group, RNs with MSDs had a higher proportion who perceived MSDs as a long-term, chronic medical condition (78.1% vs 20.7%; *p* < 0.001), being currently on medication (49.4% vs 14.7%; *p* < 0.001), using pain relief medication almost every day (9.0% vs 1.9%; *p* < 0.001), experiencing sickness absence (15.7% vs 1.1%; *p* < 0.001), seeking medical specialist consultations (odds ratio, OR 2.2; 95% CI: 2.0–2.3; *p* < 0.001), and seeking alternative medications (OR 2.5; 95% CI: 2.3–2.7; *p* < 0.001).

**Conclusions:**

Musculoskeletal disorders affected almost half of the RNs in Thailand annually. They placed a major healthcare burden and were a major cause of working days lost due to sick leaves, diminished productivity and quality of patient care. More attention should be paid to the prevention and effective management of MSDs in RNs in Thailand. Further study on ergonomics related to MSDs and its prevention are needed.

## Background

Musculoskeletal disorders (MSDs) are a major public health problem around the world [1]. In the Asia-Pacific region, about 12% to 45% of the general population suffer from chronic pain attributed to MSDs [2]. Health personnel are among those at higher risk, with rate of MSDs four times higher than workers in the manufacturing sector [3]. Nurses in particular are at high risk of MSDs due to their physically demanding jobs. A review of studies in developing countries reported that the MSDs in general workers were 37% whereas it was as high as 92% in the nursing profession [4]. However, the 12-month prevalence of MSDs in the nurse population varies widely (from 34% to 88%) [3, 5–7], with a median annual prevalence of 45% [8, 9]. The MSDs are also the main reason for sickness absence in Greek and Dutch nurses (17% and 15%) [10], medication administration errors (88%) and low productivity [11]. In Asia, the annual prevalence of MSDs among nurses ranged from 41 to 92% [4, 12–15]. Despite this very high prevalence, there is limited information on MSDs among the nursing workforce.

In Thailand, diseases of the musculoskeletal system and connective tissue is the fourth leading cause of public hospital outpatient visits (based on routine administrative health information systems), with a significant increase from 11.6 million (204 per 1000 population) in 2006 to 17.9 million (310 per 1000 person) in 2010 [16]. The MSDs are also the major cause of temporary disability among workers, contributing to 80.9% of all causes of occupational injury [17]. However, there are few studies investigating MSDs in Thai nurses, none at a national level. Of those that have been conducted, the 12-month MSDs prevalence was reported to range from 61.5% to 91.7% [18–20].

Little is known about MSDs among Thai nurses including the magnitude of the problem, and the impact on well-being. Nurse responsibilities involve patient handing activities including ergonomic factors such as lifting, awkward working postures, and pushing or pulling, activities that lead to increased risk of MSDs, especially back complaints. Ergonomic related MSDs could be minimized or prevented using ergonomic devices which are not widely used at present due to both organizational and individual factors. Organizational factors that present barriers to ergonomic device use include lack of time, lack of a policy of mandatory lift usage and employee-to-ergonomic device ratio [21], lack of knowledge and perceived needs.

To date, no study of MSDs among Thai registered nurses has been conducted. This paper estimates the prevalence of MSDs among Thai RNs based on a large, nationally representative sample of RNs, its severity, risk factors and impacts such as sickness absence and health services utilization.

## Methods

### Study design

This study utilized data from the Thai Nurse Cohort Study (TNCS) database aiming to investigate the magnitude of musculoskeletal disorders (MSDs) among registered nurses (RNs) in Thailand as well as their severities regarding sickness absence and healthcare burden. The TNCS was designed as a 20-year longitudinal cohort study. It started its first wave survey in 2009, and will survey all cohort members every two years. It aims to investigate the workforce dynamics and health conditions of Thai RNs. A nationally representative and stratified random sample of RNs holding nursing licenses granted by the Thai Nursing and Midwifery Council (TNMC) as of 2008, were surveyed using 13-page self-administered questionnaires mailed through the post. A second reminder was given to all non-respondents via a phone call and e-mail after two months following the first sending out of the questionnaire. A third and last reminder was sent one month after the 2nd reminder. The RNs who completed the questionnaires, including the signed and dated consent forms, were enrolled as members of the TNCS cohort.

### Response to questionnaire

**Fig. 1:**
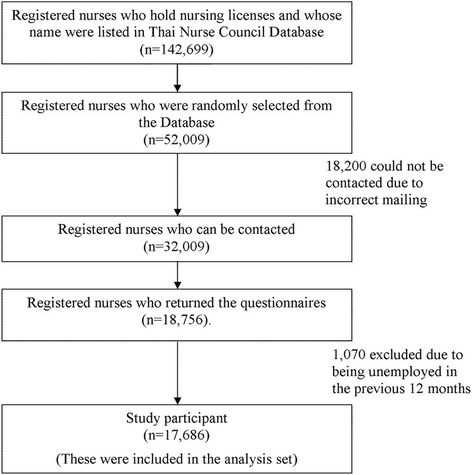
The inclusion flow chart

A total of 142,699 registered nurses who hold nursing licenses and whose names were listed in the Thailand Nursing and Midwifery Council Database, 2008 formed the population of the study. A sample of 50,209 was randomly selected based on stratified random sampling technique with probability proportional to number of nurses in each 10-year age stratum. The questionnaires were then mailed to all of them. Of these, 18,200 were returned due to incorrect mail addresses. From the remaining 32,009 that received the questionnaires, 18,756 (58.6%) responded. We excluded 1070 RNs due to being unemployed in the previous 12 months, therefore only the information of 17,686 RNs were analysed (Fig. 1).

### Measurements

The content of questionnaires was used in current study included two main sections which were baseline characteristics (birth date for computing age, gender, body weight, height, working status, type of workplace, duration of working, type of shift work, and major nursing responsibility, and other burdens); history and current illness. Reliability of 0.81 was calculated by Cronbach’s alpha and validity was operated by the ten experts to tailor questionnaires.

### Dependent variable

The Primary outcome was MSDs in Thai RNs who reportedly were having or had experienced problems involving muscles, skeletons, and joints during the previous 12 months, included: perceptions on MSDs as their chronic medical condition, hospital admission, medical conditions, seeking medical specialist consultations, seeking alternative medication, and treatment. Adverse impacts such as sick leave and healthcare burdens were also of concern and highlighted in this paper.

### Statistical analysis

For baseline characteristics, all categorical variables were reported as number and percentage. Mean and standard deviation were used to describe continuous variables. These were presented for both overall and subgroups of RNs based on different major responsibilities, namely: service, research, and administrative nurses. A 12-month prevalence of MSDs and its 95% confidence interval (95% CI) were estimated based on normal approximation to binomial distribution and were presented for both the overall and by subgroup of the RNs. Chi-square test for trend was used to test whether the prevalence increased with age, body mass index, and working duration. Percent distributions of disease burden-related characteristics were compared between those who had and did not have MSDs using chi-square tests. Odds ratio and its 95% CI were estimated for MSDs as a relative comparison with the non-MSD groups. All statistical tests were two-sided where a *p*-value of less than 0.05 was considered statistically significant. All analyses were performed using STATA version 13 (Stata Corp, College Station, TX).

## Results

### Baseline characteristics

Of the 17,686 registered nurses, their mean age was 43.5 ± 9.6 years with an average duration of working of 21.5 ± 9.9 years (Table 1). Almost all were female (97.3%), worked in hospitals (81.1%), and were government officers (82.0%). About half worked the day shift (50.7%). Almost all worked continually during their 8 h per shift (97.3%). These characteristics were similar across the different types of main task for nurses, except for those who were responsible for administration, who were older, had a longer working duration and mostly worked the day shift when compared with the service nurses.

**Table 1:** Baseline characteristics of the registered nurses presented as percentage unless specified otherwise

Characteristics	Overall (*n* = 17686)	Subgroup of registered nurses		
		Service (*n* = 13172)	Research (*n* = 1187)	Administrative (*n* = 3327)
Age (years)				
20–29	11.3	14.4	6.2	0.6
30–39	24.0	29.0	19.0	5.6
40–49	36.1	36.0	33.9	37.2
50–59	26.7	19.6	35.4	51.9
60 or greater	2.0	1.0	5.6	4.6
Mean ± SD	43.5 ± 9.6	41.4 ± 9.4	46.5 ± 9.4	50.6 ± 6.6
Gender				
Female	97.3	97.2	96.4	98.0
Male	2.7	2.8	3.6	2.0
Body mass index (kg/m^2^)				
Lower than 18	4.2	4.9	2.9	1.8
18–24.99	74.8	76.0	71.1	71.3
25 or greater	21.0	19.1	26.0	26.9
Mean ± SD	23.4 ± 3.4	22.7 ± 3.5	22.5 ± 3.5	23.3 ± 3.6
Working status				
Government officers	82.0	82.1	77.9	83.2
Government employees	3.9	4.7	3.9	1.0
State enterprise employees	0.5	0.5	0.3	0.6
Private employees	7.7	7.2	9.6	8.8
Business owners	0.6	0.6	0.5	0.6
Others	5.3	4.9	7.8	5.8
Workplace				
Hospital	81.1	84.9	26.0	86.1
Health centre, clinic	8.9	10.6	5.8	3.1
Nursing room at educational institutes	0.5	0.6	0.4	0.4
Nursing college or university	3.2	0.4	38.3	1.6
Department, division, ministry	2.6	0.9	18.9	3.4
Others	3.8	2.7	10.6	5.5
Duration of working (years)				
10 or smaller	14.5	18.6	7.7	1.0
11–20	27.0	31.9	23.3	8.8
21–30	36.3	33.6	37.5	46.3
31 or greater	22.3	15.9	31.5	44.0
Mean ± SD	21.5 ± 9.9	19.5 ± 9.7	24.5 ± 9.4	28.7 ± 6.7
Duration of shift work				
8 h per shift	97.3	97.2	95.4	97.8
12 h per shift	2.7	2.8	4.6	2.2
Type of shift work				
Most of daytime shift	50.7	47.6	40.1	66.6
Most of evening shift	5.7	7.3	1.5	0.9
Most of night shift	2.5	3.3	0.4	0.2
All three-shift rotation	20.6	27.0	3.3	1.3
Regular daytime non-shift	19.1	13.9	50.2	28.8
Work leave/unemployed	1.4	1.0	4.4	2.2

*SD* standard deviation

### Prevalence of musculoskeletal disorders

**Fig. 2:**
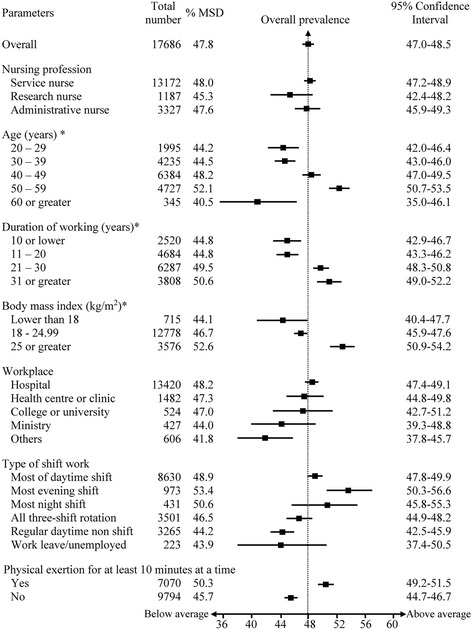
Magnitude of musculoskeletal disorders among registered nurses. MSD = musculoskeletal disorders. *Test for trend of MSD prevalence across age group (*p*-value <0.001)

The 12-month prevalence of MSDs was 47.8% (95% CI: 47.0–48.5) (Fig. 2). There was a higher prevalence of MSDs among the older aged group, those having long working duration, high body mass index, and those who worked the evening shift. The prevalence of MSDs significantly increased with age, body mass index, and working duration (P_test for trend_ < 0.001). Nurses who worked the evening shift had the highest prevalence of MSDs (53.4%; 95% CI: 50.3–56.6). For nurses who performed heavy physical activities for at least 10 min at a time had a high prevalence of MSDs (50.3%; 95% CI: 49.2–51.5).

### Severity and healthcare burden from MSDs

There were significantly (*p* < 0,001) higher proportions of various MSD-related attributes found among RNs who suffered from MSDs than those who did not. Including, perceiving MSDs as a chronic medical condition (78.1% vs 20.7%), outpatient department visit (65.7% vs 62.9%), being currently on medication (49.4% vs 14.7%), seeking medical specialist consultations (44.7% vs 27.2%), seek alternative medications (35.5% vs 18.2%), hospital admission during previous 12 months (11.6% vs 9.0%), and using pain relief medication almost every day (9.0% vs 1.9%) (Table 2). Registered nurses with MSDs were 2.2 times more likely to seek medical specialist consultations when compared with the non-MSDs group (odds ratio 95% CI: 2.0–2.3; *p*-value <0.001), and were 2.5 times more likely to seek alternative medications (odds ratio 95% CI: 2.3–2.7; *p*-value <0.001).

**Table 2:** Disease burden-related characteristics of registered nurses comparing between those who had and had no musculoskeletal disorders

Characteristics	Had MSD (*n* = 8269)		Had no MSD (*n* = 9041)		*p*-value*
	Number	(%)	Number	(%)	
Perceived the MSD as a chronic medical condition	6277	(78.1)	1819	(20.7)	< 0.001
Outpatient Department visit	5434	(65.7)	5690	(62.9)	< 0.001
Hospital admission	937	(11.6)	797	(9.0)	< 0.001
Seek medical specialist consultation ϕ	3695	(44.7)	2457	(27.2)	< 0.001
Seek alternative medications ǂ	2937	(35.5)	1644	(18.2)	< 0.001
Currently on medications	2638	(49.4)	497	(14.7)	< 0.001
Days per week of using pain relieve medications					< 0.001
None	4112	(51.3)	7306	(83.4)	
1–2 days	2593	(32.4)	1147	(13.0)	
3–4 days	589	(7.3)	146	(1.7)	
5–7 days	717	(9.0)	163	(1.9)	

*MSD* musculoskeletal disorders

*p-value based on chi-square test

ϕ Odds ratio of having musculoskeletal disorders consulted medical specialists = 2.2 (95% confidence interval: 2.0–2.3; *p*-value <0.001)

ǂ Odds ratio of having musculoskeletal disorders sought alternative medications = 2.5 (95% confidence interval: 2.3–2.7; *p*-value <0.001)

### Rate of work absence due to MSDs

Musculoskeletal disorders were the major cause of sickness absences, with an absent rate of 15.7% among RNs with MSDs in the previous 12 months whereas the absent rate from risky expose to chemical or radiation of 1.1%, and other illnesses such as sharp objects, expose to chemical or radiation, physical workplace violence, and needle stick, ranged from 0.4% to 0.5%. (Table 3).

**Table 3:** Rate of work absence due to sickness comparing between those who had musculoskeletal disorders and other type of injuries

Injuries	Total number	(% Sickness absence)
Musculoskeletal disorders	6182	(15.7)
Risky expose to chemical or radiation	4036	(1.1)
Sharp objects	4075	(0.5)
Expose to chemical or radiation	3635	(0.5)
Physical workplace violence	3652	(0.4)
Needle stick	3958	(0.4)

## Discussion

This study documented the burden of MSDs among nurses, a profession comprised mostly of women, based on a large nationally representative sample of RNs across Thailand. It was found that about half of the RNs suffered from MSDs annually, i.e., the 12-month prevalence of MSDs was 47.8%. Based on this prevalence rate, from the total of 142,699 RNs in the first wave survey in 2009, there would be roughly 68,000 nurses suffering from MSDs. Considering the sickness absence of 15.7%, there would be at least about 10,000 days lost, assuming a minimum of one-day sick leave due to MSDs. A large number of RNs who suffered from MSDs have had a considerable loss in productivity and poorer quality of patient care [11].

In addition, almost half of RNs with MSDs sought medical specialist consultations (44.7%) and were currently on medication (49.4%), particularly; about one-tenth used pain relief medications almost every day. This reflects a significant health burden faced by nurses. As pointed out by several studies, MSDs did not only affect the health of the nurses as individuals, but also contributed to adverse impacts on their quality of care for patients [11], and healthcare workforce planning due to rapid nursing turnover [22].

Musculoskeletal disorders is the leading cause of morbidity among RNs in Thailand. However, there are several other diseases and conditions commonly reported by members of the Thai Nurse Cohort. Hyperlipidemia ranked second, with an annual prevalence of 28.4%. Prevalence of others such as hypertension (7.8%), neurological diseases (5.8%), lung diseases (4.8%), hematologic diseases (4.4%), hepatitis (3.6%), cardiovascular disease (3.4%), diabetes (2.9%), and cancer (2.3%) was also reported. Therefore, it is clear that MSDs are the most significant problems, almost double that of the second ranked, hyperlipidemia. Compared to the fourth round of Thailand’s National Health Examination Survey (NHES), conducted in 2009, where the prevalence of hyperlipidemia was 19.4% among females, the prevalence of hyperlipidemia among nurses in this study (28.4%), whose majority was female, is much higher. However, there were lower prevalences of hypertension and diabetes among RNs (7.8% and 2.9%) when compared with those found in the 4th NHES among females (21.4% and 6.9% respectively) [23, 24]. The NHES, 2009 did not include MSDs; therefore, we do not have reference figures. The morbidity report of the Ministry of Public Health, 2009, reported a 29% prevalence of MSD among outpatients in public hospitals nationwide except for Bangkok Metropolitan [16]. With this reference figure, the prevalence of MSDs among RN was almost 2 times higher than that of the general population. In the context of MSD patients that seek services from out-patient departments of the public hospitals nationwide, except for Bangkok, these hospitals usually have substantial numbers of patients and long wait times, therefore, when patients choose to get services from these hospitals, they may have more significant suffering from MSDs which is in need of treatment. As a result, the figures might be under reported, since those who had mild degree of MSDs might not use the services in public hospitals. In addition, the male population may have more tolerance to pains or discomforts and may not report MSDs as frequently. In contrast, nurses are very knowledgeable about health issues, especially concerning biomedical issues. Therefore, they are more likely to detect or perceive having MSDs even in very mild forms or during the initial occurrence, and such the reported prevalence may be more precise. The high magnitude of MSD problems among Thai RNs might be due to their routine patient care giving, inevitable inappropriate ergonomic practices such as awkward postures, particularly manual handling, frequent bending, lifting, repositioning of patients or objects, and other forceful movements which are contributing factors to MSDs. The morbidity rate is expected to increase with obesity, sedentary lifestyle and ageing nurse population [1].

The MSDs prevalence of 47.8% from this study is slightly higher than some previous studies. A review on 29 articles published between 1990 and 2012 revealed that the median annual prevalence of MSDs among midwives and nurses was about 45% [8, 9]. However, it was lower than those found in 3 studies conducted in Thailand 2–3 years prior to the current study- two studies were conducted in teaching supra-tertiary hospitals whereas another was in a medium-size, general hospital, where it was reported that the prevalence ranged from 61.5% to 91.7% [18–20]. One main difference is that these 3 studies used the Standardized Nordic Questionnaire (SNQ) to assess MSDs in various body areas and defined MSDs as at least one area having a sign or symptom. The SNQ more detailed questions could stimulate a better recall of MSDs for the respondents especially when it occurred in some small area, or quite a long time ago, resulting in higher prevalence. One of the studies indicated that almost all nurses experienced MSDs (91.7%) [18]. In contrast, the Thai Nurse Cohort did not use those recall stimulation questions, this might have contributed to a much lower prevalence. However, this study showed similar MSDs prevalence, slightly higher, than a prevalence identified in large hospitals in UK (34% [6] and 45% [7]) and Hong Kong (38.9% [25] and 40.6% [26]). Most importantly, this study reflected the nationally magnitude of MSDs among RNs in Thailand and to our knowledge, this is the first nurse cohort study such as this in Southeast Asia.

This is the first study addressing health problems in women who, due to their professional duties, usually perform routine work with awkward postures involving manual handling/lifting/repositioning of patients or other objects. The study showed a higher prevalence of MSDs in the nursing profession than in the general population [16]. Globally, several organizations have been established in response to this problem, including the Bone & Joint Decade Global Alliance for Musculoskeletal Health and the National Institute for Occupational Safety and Health (NIOSH). The Thai Nurse Cohort Study (TNCS) may pave the way for establishing similar organizations responsible for the better understanding and prevention of MSDs, not only for nurses, but for all other health professionals and the general public.

This study also confirmed a higher prevalence of MSDs among the elderly. This requires policy attention since the average age group of the cohort was 43.5 years and will become older. Overweightness and shift work were also found to be associated with MSDs in other studies [27, 28]. In this study, nurses who possessed the following characteristics; age older than 50 years, being overweight, working mostly during evening shifts and performing heavy physical activities for at least 10 min at a time, comprised almost half of the nurses who reported having had MSDs (7948 out of 17,686). The 12-month prevalence of MSDs among this group was higher than other groups, i.e., 51.0% vs 45.2%, *p* < 0.001.

Understanding risk factors contributing to MSDs development, balancing workplace conditions and the capability of workers is important. Ergonomic work design has been recommended as an appropriate solution for MSDs prevention. Proper working environment arrangements and training of how to apply ergonomic concepts in the workplace are the most essential MSDs preventive measures. Understanding of working requirements, flexible work schedules as well as vocational adjustments are also needed [21, 29, 30].

Major methodological considerations include the response rate and the outcome measurement, as commonly faced by many studies conducted in general population, the low rate of response [8] can be a major source of bias. The response rate of 58.6% of the Thai Nurse Cohort is considered average among other studies of this kind [8]. We investigated the distribution of demographic characteristics using the cohort information and found that there are commonalities between the responders in the cohort and the non-responders (data to be published elsewhere). In addition, missing at random might reasonably be assumed in such a large sample size of 17,686. The outcome measurements of this study were based on self-reported, mailed questionnaires and captured the events during the previous 12-month period. The self-administered questionnaire survey was designed based on the assumption that the participants are highly educated and fully aware of their own health. Thus the 12-month prevalence of MSDs reported in this study is unlikely to be distorted by these limitations, though recall bias can be a major source of under-reporting of MSDs and other conditions.

## Conclusions

Musculoskeletal disorders (MSDs) affected almost half of the RNs in Thailand annually. They placed a major healthcare burden and were a major cause of working days lost due to sick leaves, diminished productivity and quality of patient care. Attention should be given to nurses who are older than 50 years of age, overweight, working an evening shift, and dealing with heavy physical activities, for early prevention of MSDs and the potential development of physical limitations or disabilities. There is a need to monitor the consequences of MSDs which may result in increased nursing turnover or premature exit of the profession, resulting in a nurse shortage. Further study for better understanding on the ergonomics of nursing personnel, and effective prevention interventions are recommended.

## Abbreviations

95% CI: 95% confidence interval
MSD: Musculoskeletal disorders
NHES: Thailand’s National Health Examination Survey
NIOSH: National Institute for Occupational Safety and Health
OR: Odds ratio
REQW: Board Committee of Research and Training Centre for Enhancing Quality of Life of Working Age People
RN: Registered nurses
SNQ: Standardized Nordic Questionnaire
TNCS: Thai Nurse Cohort Study
TNMC: Thai Nursing and Midwifery Council
